# Thermal Effects on Dental Pulp during Laser-Assisted Bleaching Procedures with Diode Lasers in a Clinical Study

**DOI:** 10.3390/jcm13082301

**Published:** 2024-04-16

**Authors:** Marlene Petersen, Andreas Braun, Rene Franzen

**Affiliations:** Department of Operative Dentistry, Periodontology and Preventive Dentistry, RWTH Aachen University, 52074 Aachen, Germany; marlene.petersen@rwth-aachen.de (M.P.); franzen@aalz.de (R.F.)

**Keywords:** dental pulp, endodontics, lasers, temperature, tooth bleaching

## Abstract

**Background** In the current cosmetics industry, bleaching is often associated with lasers. However, such treatment also harbors risks. Tooth death is observed at pulpal temperature increases ≥5.6 °C. Therefore, it seems important to investigate the effects of using different lasers. The aim of this study was to determine pulpal temperature increases at different laser parameters during bleaching by modeling a realistic environment and to compare the temperature recording using a thermocouple and thermal camera. The authors assumed that there are laser settings for the lasers used at which the pulpal temperature increases are <5.6 °C and that the temperature recordings with thermocouples and thermal cameras differ only minimally. **Methods** Human teeth were used, which were extracted for dental reasons. During experiment, teeth were bleached conventionally and by laser activation at 940 nm, 445 nm, and 970 nm. The temperature in the pulp was recorded using thermocouples. In a second setup, longitudinally halved teeth were bleached, while the temperature in the pulp was recorded with a thermocouple and thermal camera. Descriptive statistics were used. The significance level is 0.05. **Results** In addition to conventional bleaching, temperature increases <5.6 °C were observed for bleaching at 940 nm 1.5 W, at 445 nm 0.3 W, and at 970 nm 0.5 W. For bleaching procedures using 940 nm 7 W, 940 nm 2 W, 445 nm 0.5 W, and 970 nm 1 W, the temperature increase was ≥5.6 °C. Significant differences (*p* < 0.05) were found in the maximum temperature increases (°C) between all groups. Temperature recordings using a thermocouple and thermal camera differed by about 2.3 °C. The working hypotheses were confirmed. **Conclusion** With laser bleaching, attention must be paid to the type of laser, its power, and the time in order to avoid excessive overheating of the dental pulp.

## 1. Introduction

As a bright, white-toothed smile has become one of today’s beauty ideals, there are more and more people wishing to undergo tooth whitening. The active component in this procedure is hydrogen peroxide, which releases free radicals when it decomposes. Oxygen radicals cause oxidation of the dyes, resulting in tooth whitening. Activation of bleaching gel, e.g., by heat, light, or laser irradiation, accelerates the bleaching process by catalyzing the dissociation of the hydrogen peroxide [1].

In general, whitening techniques must be considered from a clinical medical point of view. Tooth whitening, especially repeated whitening, carries the risk of causing structural changes to the enamel and exposed root dentin, leading to hypersensitivity and future brittleness, resulting in fractures and breaks. Exposed dentinal tubules harbor the risk of free radical migration into the pulp [2,3,4,5,6,7]. During bleaching processes in combination with hydrogen peroxide, teeth can show reduced cell viability [8], altered cell morphology [9], and increased expression of inflammatory mediators (e.g., interleukin-1 and tumor necrosis factor) [8,10]. Regression of the inflammation can be observed after 30 days [11,12]. Tissue repair initiates tertiary dentin formation [11]. In laser bleaching, the laser acts like a catalyzer [1]. The reaction speed of the chemical reaction is increased by lowering the activation energy of the reaction. This allows the hydrogen peroxide to lie less, which minimizes the risks to tooth enamel, dentin and dental pulp. In addition, bleaching processes can also affect dental restorations. Reduced microhardness, slight roughening and the formation of cracks can be observed [13]. In addition, the active substances used can also cause changes in the neighboring tissues in the oral cavity. Even the effect on distant structures such as the gastric mucosa cannot be excluded if the tooth whitening agent comes into direct contact with the mucosa, for example by swallowing [14].

Activation of bleaching gel can lead to an increase in pulpal temperature. These temperature increases depend on numerous factors, such as the energy-applying medium, the set parameters, or the tooth type [15]. Nyborg et al. found that heat can result in the loss of odontoblasts and the demise of pulpal tissue [16]. Irreversible pulpal damage occurs at pulpal temperature increases ≥5.6 °C [17]. The bleaching agent plays a protective role here by reducing the temperature increase in the pulp by up to 30% [18]. Blood circulation in the pulp also reduces the temperature increase in the pulp chamber caused by bleaching [19]. In addition, laser bleaching has the advantage that the laser beam can be completely absorbed by the bleaching agent used, which protects the vitality of the teeth. In this way, the laser beam does not penetrate the hard tissue of the tooth so that no direct heating of the tooth structure occurs [20].

As part of the modernization of dental techniques, lasers are increasingly being used in dental treatments [21,22,23,24,25,26,27]. Of particular relevance to endodontics is the limited exploration of various lasers in tooth bleaching and the insufficient investigation into how the heat generated during laser irradiation may affect the dental pulp [3,28,29]. Given the potential risk of a physiologically undesirable increase in pulp temperature, which could lead to irreversible pulpitis or pulp necrosis [17]—thus requiring endodontic treatment—it is imperative to avoid such outcomes. Preserving the vitality of teeth is paramount when pursuing tooth whitening, aligning with the contemporary cosmetic preference for light-colored teeth.

The pulp reacts to an increase in temperature with an increase in blood flow [30]. The blood flow reduces the temperature increase in the pulp chamber caused by heat. The higher the flow rate, the lower the temperature rise [19]. The pulp therefore has its own protective mechanism against temperature increases. The blood flow should consequently not be neglected.

As lasers are increasingly being used in dentistry, their risks need to be investigated. The aim of this study is to find laser settings in which the temperature increase in the pulp is <5.6 °C. Temperature increases ≥5.6 °C lead to irreversible pulpitis [17]. To date, there are only two studies in the literature in which the temperature changes of the pulp during laser-assisted tooth whitening with a diode laser with a wavelength of 445 nm have been investigated [31,32]. The 2023 study found that an average power of a diode laser (445 nm) of 0.5–2 W and an irradiation time of 10–60 s can be considered safe for pulp health when a red-colored bleaching gel is used [31]. The 2021 study found that the temperature rise of the pulp after bleaching with the diode laser was within the safe range at a wavelength of 970 nm and a power of 1.5 W and 2 W and at 445 nm and a power of 1 W and 1.5 W. The highest temperature rise of the pulp was observed with the diode laser at a wavelength of 970 nm and a power of 2 W (2.0 ± 0.5), while the lowest temperature rise of the pulp (0.1 ± 0.2) was observed with the diode laser at a wavelength of 445 nm and a power of 1 W [32]. Due to the high absorption in hemoglobin and melanin [33], a laser wavelength of 445 nm is mainly used in oral surgery [34]. As pulp tissue is rich in hemoglobin, most of the light energy would be absorbed there when the laser beam is directed at the tooth. Therefore, the bleaching gel should be designed to absorb the laser energy at the tooth surface and thus prevent a sharp increase in pulp temperature.

The present study attempts to come as close as possible to the in vivo conditions; in addition to the body temperature setting, a blood flow simulation is introduced and also taken into account, unlike in the studies mentioned above [31,32]. Furthermore, the teeth are not directly immersed in a temperature-controlled water bath as in the study of Papadopoulou et al. [31] but are embedded in a resin to simulate the thermal properties of bone. This creates an environment in which a lower, more realistic, thermal conductivity occurs. This would lead, in theory, to higher intra-pulpal temperatures and, hence, provide a more critical approach to the research question. In addition, different types of teeth are used, the lasers differ in their power, and the time and duration of the temperature recording was chosen differently. The studies clearly differ in their methodology. The null hypothesis to be refuted states that there are only laser settings for the diode lasers used at which the pulpal temperature increases measured in vitro are not below the value of 5.6 °C, which is considered critical. The authors assume that, taking into account the laser types, their power, and the time, laser settings can be found at which the pulpal temperature increase is <5.6 °C.

## 2. Materials and Methods

The present study is a clinical study whose basic principle is to investigate the safety and effectiveness of laser bleaching.

The teeth used were collected from various dental practices in the state of North Rhine–Westphalia, originated from women and men of different ages, and had been extracted for dental (especially periodontal) or orthodontic reasons. Only mature human anterior teeth, canines, and premolars were included that did not have any vestibular fillings and/or carious lesions, with intact roots, and with the crown pulp not obliterated. This study was conducted in accordance with the Declaration of Helsinki and the ethical harmlessness of using surplus, anonymized biomaterial was approved by the Ethics Committee of Medical Faculty of the RWTH Aachen (EK 141/21). The teeth were donated for research purposes. Due to the classification of the teeth as medical waste and the given anonymity, the signed consent of the tooth donors was waived. After the teeth had been extracted and the dentist had informed the patients about the further use of the teeth, the teeth were placed in pure demineralized water. Storing in pure demineralized water prevents minerals from accumulating in the teeth, which could impair the whitening process. For the first experimental setup, 25 teeth were used. For the second experimental setup, 6 were used.

First, the teeth were shortened by 2 mm starting from the apex (Cut-grinder Primus, Walter Messner GmbH, Oststeinbek, Germany). After shortening, the teeth were prepared manually and mechanically from the apex (K-file 25 mm, ISO 15 and 20, VDW GmbH, Munich, Germany; FG 249C014, Hopf, Ringleb & Co. GmbH & Cie., Berlin, Germany; Gates Glidden drill, Ref. 212, Dentsply Maillefer, Ballaigues, Switzerland) and rinsed with 3% sodium hypochlorite (NaOCl; Hedinger, Stuttgart, Germany) and 17% ethylenediaminetetraacetic acid (EDTA; Lege Artis Pharma GmbH + Co. KG, Dettenhausen, Germany). Before being prepared for the respective experimental setup, the teeth were cleaned using a scaler (Hu-Friedy Mfg. Co., LLC, Chicago, IL, USA), pumice powder (Ref 100360, Ernst Hinrichs Dental GmbH, Goslar, Germany), and polishing brushes (No. 835, Kerrhawe SA, Bioggio, Switzerland).

### 2.1. Investigation of the Thermal Effect

**Figure 1:**
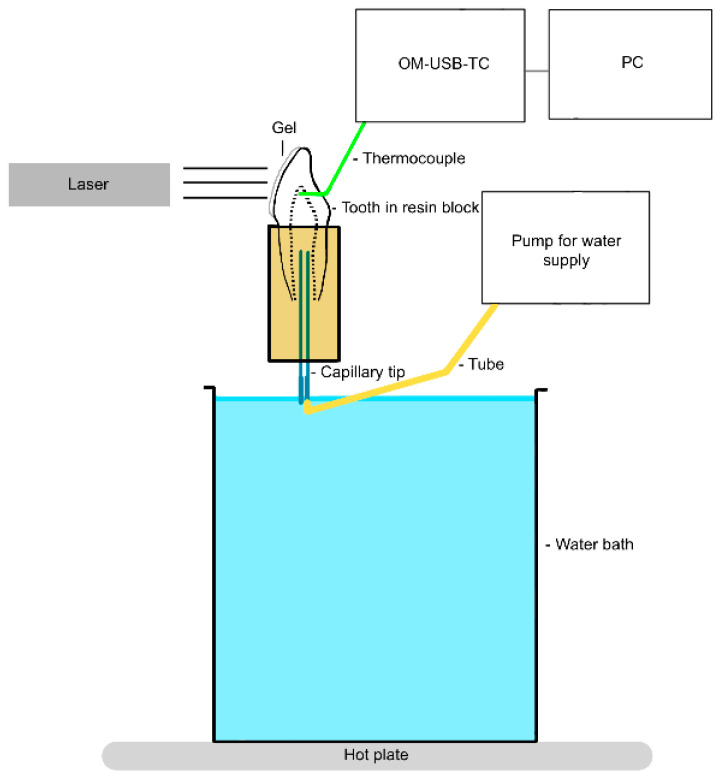
Diagram of the first experimental setup, lateral view (created with GoodNotes 4, Hongkong, China); OM-USB-TC = USB measurement system (OMEGA Engineering, Norwalk, CT, USA).

**Figure 2:**
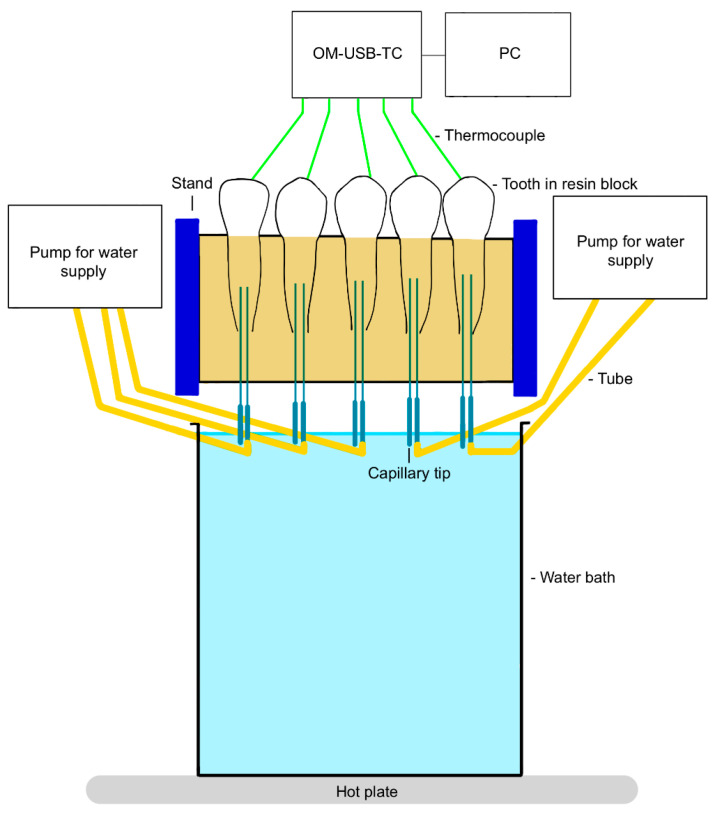
Diagram of the first experimental setup, front view (created with GoodNotes 4, Hongkong, China).

To investigate the thermal effect (first experimental setup; Figure 1 and Figure 2), the shortened and cleaned teeth were connected via capillary tips (No. 186, Ultradent Products Inc., South Jordan, UT, USA) from the apex to a tubing system (PharMed^®^ BPT, SCO318, IDEX Health & Science Ismatec^®^, Wertheim, Germany) designed to simulate blood circulation [19].

The tubing was connected to previously calibrated pumps (Ismatec^®^ Reglo ICC, Cole-Parmer GmbH, Wertheim, Germany). Since the pulpal blood flow rate is estimated to be in the range of 20–60 mL/min per 100 g [35] and a third molar has a pulpal weight of 13.10 mg [36], 5.93 µL/min approximates the blood flow rate in the pulp. Demineralized water was pumped through the tubes and the pulp chamber at a constant flow rate of 5.93 µL/min.

Five teeth (central incisor, lateral incisor, canine, first premolar, and second premolar) were embedded in a polyurethane block (ISO-PUR K 760 Beige, Batch: GE 0613, Mewa Electronic GmbH & Co. KG, Pinneberg, Germany) to represent a quadrant of the jaw. Bone has a thermal conductivity of 0.58–1.2 W/(mK), a density of 1.36 kg/cm^3^, and a heat capacity of 1.6–2.1 kJ/(kgK). As polyurethane is very similar to bone in these properties, it is ideal for simulating bone [37]. The mixing ratio chosen was 6:1.

**Figure 3:**
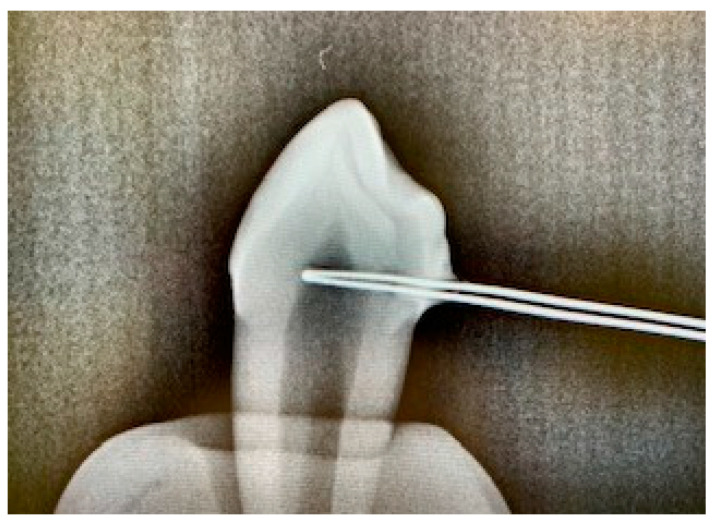
X-ray control image to assess the correct position of the thermocouple.

For temperature recording, a thermocouple (5TC-TT-KI-30-1M, OMEGA Engineering) was inserted into the tooth crown via a drilled hole (FG 837KRF 016, Edenta AG, Au, Switzerland; Rose Drill Iso 310 204 001001 009, Hager & Meisinger GmbH, Neuss, Germany). This thermocouple was inserted in such a way that it came to rest directly on the vestibular pulp chamber wall, touching it. The correct position of the thermocouple was confirmed by X-ray (Figure 3).

Body temperature (36.5 ± 1.5 °C), which was confirmed via the thermocouples, was set before data collection. The setting was made using the heated water bath. The thermocouples were connected to a computer via a USB measurement system (OM-USB-TC, OMEGA Engineering). The InstaCal 6.1 software program (Measurement Computing Corporation, Norton, MA, USA) was used to test and calibrate the thermocouples. Measurements were made using the TracerDAQ 2.2 software program (Measurement Computing Corporation).

The thermal effect was investigated in the following test groups, each of them consisting of 15 teeth. The laser settings are determined by preliminary tests and studies [28,31,32] or by following the manufacturer’s instructions:

- Group 1 (control): no laser activation, bleaching gel (Perfect Bleach Office+, VOCO GmbH, Cuxhaven, Germany), and resting time 15 min.
- Group 2 (test): diode laser EzLase 940 nm 7 W (BIOLASE, Inc., Foothill Ranch, CA, USA), bleaching handpiece (EzLase* Whitening Handpiece; BIOLASE, Inc., Foothill Ranch, CA, USA), bleaching gel (Laserwhite*20 Tooth Whitening Gel Kit; BIOLASE, Inc. Aachen, Germany), irradiation time 30 s, 1.5 min rest phase, repeat irradiation, resting time 15 min, distance between tooth and laser approx. 1 mm, and 105 J/cm^2^ of applied energy (two cycles).
- Group 3 (test): diode laser EzLase 940 nm 2 W, single fiber 300 µm (EzTip E3–9 mm, BIOLASE, Inc.), bleaching gel (Laserwhite*20), irradiation time 30 s, repeat irradiation, resting time 15 min, distance between tooth and laser approx. 1.5–2.0 cm, spot size on tooth: 154–255 mm², and 47–78 J/cm^2^ per tooth (two cycles).
- Group 4 (test): diode laser EzLase 940 nm 1.5 W, single fiber 300 µm, bleaching gel (Laserwhite*20), irradiation time 30 s, repeat irradiation, resting time 15 min, distance between tooth and laser approx. 1.5–2.0 cm, spot size on tooth: 154–255 mm², and 35–58 J/cm^2^ per tooth (two cycles).
- Group 5 (test): diode laser SiroLaser Blue 445 nm 0.5 W (Dentsply Sirona, Charlotte, NC, USA), single fiber 320 µm (Dentsply Sirona), bleaching gel (Perfect Bleach Office+, VOCO GmbH, Cuxhaven, Germany), irradiation time 30 s, resting time 15 min, distance between tooth and laser approx. 1.5–2.0 cm, spot size on tooth: 20–28 mm², and 53–77 J/cm^2^ per tooth (one cycle).
- Group 6 (test): diode laser SiroLaser Blue 445 nm 0.3 W, single fiber 320 µm, bleaching gel (Perfect Bleach Office+), irradiation time 30 s, resting time 15 min, distance between tooth and laser approx. 1.5–2 cm, spot size on tooth: 20–28 mm², and 32–46 J/cm^2^ per tooth (one cycle).
- Group 7 (test): diode laser SiroLaser Blue 970 nm 1 W (Dentsply Sirona), single fiber 320 µm, bleaching gel (Perfect Bleach Office+), irradiation time 30 s, resting time 15 min, distance between tooth and laser approx. 1.5–2.0 cm, spot size on tooth: 20–28 mm², and 106–153 J/cm^2^ per tooth (one cycle).
- Group 8 (test): diode laser SiroLaser Blue 970 nm 0.5 W, single fiber 320 µm, bleaching gel (Perfect Bleach Office+), irradiation time 30 s, resting time 15 min, distance between tooth and laser approx. 1.5–2.0 cm, spot size on tooth: 20–28 mm², and 53–77 J/cm^2^ per tooth (one cycle).

Each group was tested on 15 different teeth, i.e., on three quadrants of a jaw. The gel was applied successively—starting with the central anterior tooth and ending with the second premolar—to the vestibular surface of the teeth in a thin layer of about 1–2 mm. Each tooth was irradiated individually, starting with the central incisor, using the laser handpiece with a fiber tip in noncontact mode. The irradiation was repeated after the last tooth—i.e., the second premolar—had been irradiated. When the bleaching handpiece was being used, all of the teeth were bleached simultaneously. The resting time of 15 min started after the second premolar had been completely irradiated. At the end of the 15 min resting time, the gel was rinsed off under running water.

### 2.2. Method Comparison

**Figure 4:**
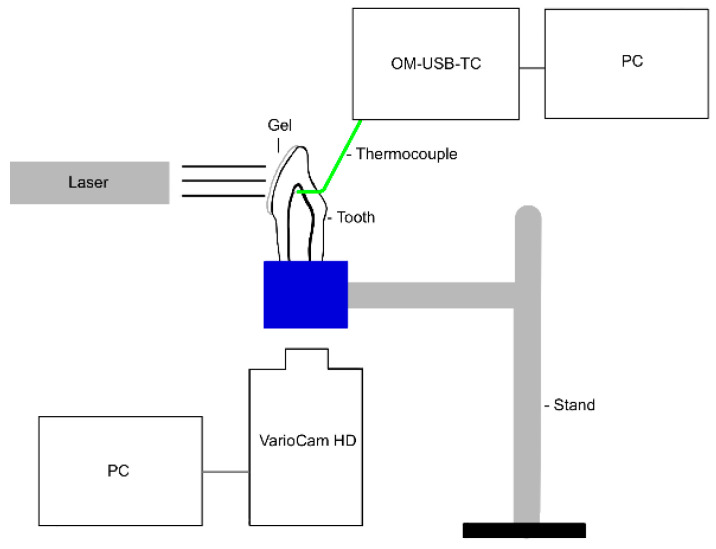
Diagram of the second experimental setup (created with GoodNotes 4, Hongkong, China).

For method comparison (second experimental setup, Figure 4), the shortened and cleaned teeth were cut in half longitudinally (Cut-grinder Primus) to provide an unobstructed view of the pulp.

A hole was drilled in the vestibulo-oral direction in the upper area of the pulp chamber on the oral tooth wall (FG 249C 012, Hopf, Ringleb & Co. GmbH & Cie.) in which a thermocouple (5TC-TT-KI-30-1M, OMEGA Engineering) was placed. The temperature in the pulp was recorded by means of both a thermocouple and thermal camera (VarioCam^®^ HD Head, Jenoptik Optical Systems GmbH, Jena, Germany). A sharp image of the pulp chamber indicates correct positioning of the thermal camera. Measurements were made with two lenses (IR 1.0/30 VC3, Jenoptik; Converter 0.5X, Jenoptik) simultaneously. The thermocouple was reconnected to the computer, checked, and calibrated. The thermal camera was connected to a laptop. The measurements were made using the TracerDAQ (Measurement Computing Corporation) and Irbis 3 plus software programs (InfraTec GmbH, Dresden, Germany).

Each group was tested on five different teeth (central incisor, lateral incisor, canine, first premolar, and second premolar), with measurements repeated three times on each tooth. The gel was applied to the vestibular half of the tooth in a thin layer about 1–2 mm thick. Data collection was commenced a few seconds before gel application and continued for 2 min. These 2 min included the time for the gel application, radiation, and the resting time. Since the thermographic image of the thermal camera also captured the thermocouple, the data collection point of the thermal camera that needed to be determined was manually set at the level of the thermocouple. Comparison between the thermocouple (group 9/11/13/15) and the thermal camera (group 10/12/14/16) was carried out on the following test groups (the whitening gels and lasers correspond to those from the first experimental setup):

- Group 9/10: no laser activation and bleaching gel (Perfect Bleach Office+).
- Group 11/12: EzLase 940 nm 1.5 W, single fiber 300 µm, bleaching gel (Laserwhite*20), and irradiation time 30 s.
- Group 13/14: SiroLaser Blue 445 nm 0.3 W, single fiber 320 µm, bleaching gel (Perfect Bleach Office+), and irradiation time 30 s.
- Group 15/16: SiroLaser Blue 970 nm 0.5 W, single fiber 320 µm, bleaching gel (Perfect Bleach Office+), and irradiation time 30 s.

In order to compare methods and to test the influence of thermal paste (MX-4 Thermal Compound, Arctic GmbH, Braunschweig, Germany), the process described above was repeated for groups 9/10 and 11/12 using the thermal paste for three series of measurements per group on one tooth.

### 2.3. Evaluation and Statistics

The values recorded were analyzed using Microsoft Excel 2013 (MS Excel) (Microsoft, Redmond, WA, USA) and Statistical Package for Social Sciences 28 (SPSS) (IBM, Armonk, NY, USA).

The value to be determined in the first experimental setup is the maximum pulpal temperature increase (°C). The initial temperature is defined as the temperature at the time of the start of gel application to the tooth. Since, according to Zach and Cohen, irreversible pulp damage can be associated with a pulpal temperature increase ≥5.6 °C [17], this value is assumed to be the critical value.

Since the focus in the second experimental setup was on comparing the temperature recording methods by means of thermocouple and thermal camera, the value to be determined was the temperature difference (°C) between the two measuring methods per second.

The study mainly used descriptive statistics. The significance level is 0.05. The statistical test procedures used were ANOVA and the Tukey range test.

## 3. Results

### 3.1. Investigation of the Thermal Effect

Table 1 shows the minimum and maximum for the maximum pulpal temperature increase (°C) in each group and for the respective bleaching process. In addition, the maximum duration of the pulpal temperature increase ≥ 5.6 °C (s) is listed.

Statistical analysis showed significant differences (*p* < 0.05; *p* = < 0.001) in the maximum pulpal temperature increases (°C) between all groups during both bleaching procedures. Tukey’s range tests showed significant differences (*p* < 0.05) between the control group and groups 3 (*p* = <0.001), 5 (*p =* 0.009), and 7 (*p* = 0.009), while the differences between groups 4 (*p* = 0.785), 6 (*p* = 1.000) and 8 (*p* = 1.000) and the control group were not significant (*p* > 0.05). Significant differences (*p* < 0.05) were also found between groups 3 and 4 (*p* = <0.001), 5 and 6 (*p* = 0.022), and 7 and 8 (*p* = 0.022).

### 3.2. Method Comparison

For method comparison, the value to be determined was the Δt (°C) between the temperature recording by thermocouple and thermal camera per second (Table 2).

The mean values (Table 2) varied by 0.3 °C. The average of the mean values was 2.3 °C.

After adding the thermocouple to the corresponding heat-conducting paste, temperature fluctuations between 0.1 °C and 0.3 °C were observed. When the bleaching gel alone was used with the MX-4 thermal paste, the mean value for Δt was 2.7 °C. When the bleaching gel was activated with EzLase 940 nm 1.5 W and using the thermal paste, the mean value for Δt was 2.9 °C. The mean values listed above result in a common mean value of 2.8 °C.

## 4. Discussion

During conventional bleaching and laser bleaching with 940 nm 1.5 W, 445 nm 0.3 W, and 970 nm 0.5 W, the temperature increase of the pulp was below the critical value of 5.6 °C. Activation of the bleaching gel by laser accelerates the bleaching process [1]. One of the negative side effects of laser-activated bleaching can be an increase in pulpal temperature [15]. Zach and Cohen have shown that irreversible pulp damage (e.g., pulpal necrosis) is caused at a pulpal temperature increase ≥ 5.6 °C [17].

The temperature recordings with thermocouple and thermal camera differed by around 2.3 °C.

Increases in pulpal temperature depend on numerous factors, such as the energy-supplying medium, the parameters used, the distance between the tooth and the energy-supplying medium, the duration of irradiation, and the tooth type [15,18,38,39].

When a laser is used as an energy source in combination with a bare fiber (divergent beam), too short a distance reduces the area to be irradiated. This may result in an increased temperature rise [18].

One study detected a thermal insulation effect of dentin: the thicker the dentin, the lower the pulpal temperature increase [40]. The temperature rise in the pulp is significantly affected by the thickness of the tooth structure [41]. Incisors in the lower jaw or lateral incisors in the upper jaw, which have a low heat capacity, react more sensitively to pulpal temperature increases [29].

Opinions in the literature vary with regard to the effect of bleaching on the tooth structure. Some authors have not identified any clinically significant changes in tooth structure [3,42,43], whereas other authors have emphasized structural changes such as pronounced perikymata, erosions, loss of interprismatic substance, increases in surface roughness, and reduced microhardness [3,4,5,6,7].

In addition to reducing pulpal temperature increases by up to 30% [18], the whitening gel prevents direct heating of the tooth when the laser beam is fully absorbed. Gutknecht et al. reported that an Er:YAG laser beam (λ = 2.94 µm) is completely absorbed in aqueous gel [20]. It has also been reported that an Er,Cr:YSGG laser (λ = 2.78 µm) can be successfully used for bleaching treatment. The pulpal temperature increases measured were below 5.6 °C [44]. The wavelengths of diode lasers are absorbed particularly well in dark pigments such as beta carotene [15,39].

Braun et al. emphasized that blood circulation in the pulp reduces the temperature increase in the pulp chamber caused by the application of heat; the higher the flow rate, the lower the temperature increase [19].

An investigation of thermal effects on the pulp during a bleaching procedure with a 940 nm diode laser (bleaching handpiece, 7 W, two cycles of 30 s, resting period 2.5 min) showed a maximum temperature increase of 2.6 °C [18]. An in vivo study showed that, after laser activation of the bleaching gel with a 940 nm diode laser (400 µm fiber, 7 W, maximum total energy delivered per tooth 210 J, 1 mm distance), patients had a 100% positive vitality sample in the teeth after 1 year [28]. In 2021, a study was published that recorded the temperature increase after bleaching with different diode lasers. The highest temperature increase (2.0 ± 0.5) was obtained with a 970 nm diode laser at 2 W. The lowest temperature increase (0.1 ± 0.2) was obtained with a 445 nm diode laser at 1 W [32].

The limit for the temperature increase is based on the findings of Zach and Cohen [17]. Since their studies were carried out on rhesus monkeys, they are only transferable to humans to a limited extent. The fact that irreversible pulp damage was found in only 15% of the teeth also argues against general validity. Nevertheless, the 15% percentage figure cannot simply be ignored. The results presented by Zach and Cohen continue to be widely used in the current literature [15,29,45] and can be regarded as reference values.

In order to approach as closely as possible to common real-world everyday practice, a wide variety of teeth were used in the present study and the application of the bleaching gel and positioning of the laser were performed manually. The resulting errors described below are errors that are part of the clinical process. Due to the manual positioning of the laser, the required distance of approximately 1 mm or 1.5–2.0 cm between the laser and the teeth could not be reliably maintained. If the distance is not maintained, it can lead to an excessive temperature increase [18]. Manual application may reduce the gel’s protective effect on the pulpal temperature increase [18] if an insufficient amount is applied. Since a large variety of teeth were used, they all received identical treatment and were stored in the same conditions, avoiding further divergences. The scheduling of the irradiation using the laser settings made it impossible for the laser to irradiate the teeth for too long. Dark pigmented bleaching gels were used for absorption of the laser beam in the bleaching gel [15,20,39].

Since blood circulation in the pulp is known to be beneficial for maintaining tooth vitality, by reducing the temperature rise caused by bleaching [19], constant blood circulation was simulated by means of capillary seats. The dynamics of human blood flow [30] could not be taken into account in this experimental setup. To simulate blood circulation, the root canal was prepared widely. The resulting loss of mass could lead to a loss of the heat-insulating effect of the dentin [40].

Like any other measuring instrument, the thermocouple also has a degree of measurement inaccuracy that cannot be neglected. External influences also need to be taken into account. When temperature changes during the bleaching processes are observed, it is noticeable that some Δt curves cool down immediately after gel application and fall below the body temperature set at the beginning during the entire measurement process. The reason for this phenomenon is not clear, although some explanations may be suggested. On the one hand, too much gel could have been applied due to the manual gel application. The more gel applied, the more heat is removed from the tooth. Secondly, the teeth might be small teeth with a smaller tooth structure. The smaller the tooth, the stronger the cooling effect. Another possibility would be a large pulp chamber. The larger the pulp, the more demineralized water there is in it. Water has a large heat capacity and, therefore, cools the tooth more. A combination of all three of the above explanations is also possible.

Investigations of the thermal effect of different lasers on the pulp [18,28,32,38] cannot be confirmed by the results obtained here. However, direct comparison is not possible due to differences in methodology. The methodology used here was an attempt to approach as closely as possible to conditions in everyday practice and, at the same time, to reproduce in vivo conditions.

Laser bleaching is increasingly being used. The associated risks must be analyzed in order to avoid them. Irreversible pulpitis must be avoided as this would lead to root canal treatment. As this study is an in vitro study, further studies must concentrate on the laser parameters available here and substantiate them. In addition, the blood flow in particular should be taken into account. Options for lowering the temperature after laser irradiation should also be discussed. For example, cooling the tooth after bleaching could be considered.

The temperature recordings carried out here using a thermocouple and a thermal camera differed by about 2.3 °C without thermal paste and by about 2.8 °C with thermal paste. Since the same framework conditions were created for both temperature recording techniques, with the temperature recordings being carried out in parallel, it is possible to use both temperature recording techniques, taking the common mean value into account. The mean value is probably the result of deviating calibrations of the two measuring instruments and/or their measurement inaccuracy. In addition, minimal movements in the setup cannot guarantee that the thermal camera’s previously defined measuring point corresponds to that of the thermocouple. In addition, taking several series of measurements into account that differed in the tooth being used, the bleaching gel, and the distance between the tooth and the laser could also have an influence on the mean value.

It is not advisable to use a thermal camera to record the temperature inside a tooth, i.e., in the pulp chamber. A thermal camera is only able to record the surface temperatures. In order to record the pulp temperature, the tooth would have to be cut in half lengthwise. Cutting the tooth in half allows a clear view of the pulp. In addition to the clear view of the pulp, halving the tooth also reduces its heat capacity. The same energy input would lead to a higher temperature increase. It also makes it more difficult to simulate blood flow and generate body temperature.

## 5. Conclusions

The positive cosmetic effect of tooth whitening is offset by several risks. In addition to the main risk of hypersensitivity, an increase in pulp temperature ≥5.6 °C can lead to irreversible pulpitis. In the present study, in addition to conventional bleaching, temperature increases <5.6 °C were observed for bleaching at 940 nm 1.5 W, at 445 nm 0.3 W, and at 970 nm 0.5 W. Temperature recording using a thermocouple and thermal camera is possible when the common average value is taken into account. Thermal paste does not appear to have any influence here.

Further clinical studies should focus on the efficacy of tooth whitening treatments with the laser parameters presented here in order to substantiate the laser parameters determined here, as well as other non-laser-related influences. The limitations of this in vitro study must be taken into account: Only pulpal temperature changes were investigated in this study. Structural changes or enamel and pulp reactions were not considered. It is further to be considered that, beside the thermal risk to the pulp, other risks, such as chemical influences, exist as well and have not been part of this study. Until fundamental questions of pathobiological and clinical safety are not tested according to all relevant international regulations, no clinical study can be proposed on the outcome of the presented in vitro study. In general, the patient and the dentist should weigh up the benefits and risks of bleaching. In this case, one benefit is outweighed by several risks.

## Figures and Tables

**Table 1:** Results of the first experimental setup.

Group	Minimum of Maximum Pulpal Temperature Increase in Degrees Celsius (°C)	Maximum of Maximum Pulpal Temperature Increase in Degrees Celsius (°C)	Maximum Duration of the Pulpal Temperature Increase ≥5.6 °C in Seconds (s)
1	0	4.3	
2	1.0 (first bleaching procedure) 0.8 (second bleaching procedure)	4.7 (first bleaching procedure) 6.4 (second bleaching procedure)	12
3	3.4 (first bleaching procedure) 2.5 (second bleaching procedure)	8.2 (first bleaching procedure) 11.3 (second bleaching procedure)	132
4	0 (first bleaching procedure) –2.1 (second bleaching procedure)	5.4 (first bleaching procedure) 5.5 (second bleaching procedure)	
5	0.4	5.6	19
6	0	4.1	
7	0	9.2	58
8	0	4.6	

The minimum and maximum for the maximum pulpal temperature increase (°C) in each group and for the respective bleaching process and the corresponding maximum duration of the pulpal temperature increase ≥5.6 °C in seconds.

**Table 2:** Results of the second experimental setup.

Group	Minimum of Temperature Differences in Degrees Celsius (°C) per Second	Maximum of Temperature Differences in Degrees Celsius (°C) per Second	Average of Temperature Differences in Degrees Celsius (°C) per Second
9/10	0.3	3.2	2.2
11/12	0.3	3.7	2.2
13/14	0.9	4.9	2.5
15/16	1.1	3.6	2.4

The minimum and maximum temperature differences (°C) per second for each group in comparison of the temperature recording by means of thermocouple and thermal camera, and the respective resulting average of temperature differences (°C) per second.

## Data Availability

The data presented in this study are available on request from the first author (M.P.).
